# Accessibility of Ontario pharmacies offering COVID-19 vaccination by rurality, community material deprivation, and ethnic concentration: a repeated cross-sectional geospatial analysis

**DOI:** 10.1186/s12889-025-24929-w

**Published:** 2025-11-12

**Authors:** Mhd Wasem Alsabbagh, Markus Wieland, Shayna Pan, Nancy M. Waite, Sherilyn K.D. Houle, Kelly Grindrod

**Affiliations:** 1 School of Pharmacy, Faculty of Science, University of Waterloo, 10A Victoria St. S, Kitchener, N2G 1C5 ON Canada; 2 University of Waterloo (Geospatial Centre), Kitchener, ON Canada

**Keywords:** Pharmacy, COVID-19 vaccine, Vulnerability, Community, Accessibility

## Abstract

**Background:**

Community pharmacies are largely recognized as geographically accessible; yet concerns arise regarding inequitable access to COVID-19 vaccination, especially during early vaccine availability.

**Objectives:**

This study aims to investigate the geographic accessibility of community pharmacies offering COVID-19 vaccination in Ontario’s from April to December 2021 considering community-level rurality, material deprivation, and ethnic concentration.

**Methods:**

Data from the Ontario Ministry of Health website COVID-19 vaccination pharmacies between April 27, 2021 and December 20, 2021, were analyzed. Pharmacy addresses were geocoded using Environics Analytics Business Data and the Postal Code Conversion File (PCCF+). Material deprivation and ethnic concentration at the Dissemination Area (DA) level were based on Public Health Ontario’s marginalization data and organized into quintiles. Mean geographic accessibility was calculated for each quintile using the 2-Step Floating Catchment Area method using service areas of 1,000, 1,500, or 3,000 m for urban DAs and 10,000 m for rural DAs. Analysis of Variance (ANOVA) was used to compare mean geographic accessibility across eight selected dates reflecting vaccine eligibility and availability changes.

**Results:**

Of 15,174 pharmacies identified, 92.9% were successfully linked to geographic coordinates. Three eras of vaccine availability were identified: [1] Intermediate; [2] Scarcity (May 2021); and [3] Abundance (November and December 2021). During vaccine shortages, more deprived and ethnically concentrated urban areas had greater geographic accessibility than less deprived areas, while rural areas had no access. For example, during vaccine scarcity, urban DAs in the highest ethnic concentration quintile had an accessibility score of 6.55 compared to 0.18 in the lowest quintile. During other periods, more deprived urban areas either showed higher geographic accessibility or no significant difference compared to less deprived areas; however, rural deprived areas generally had lower geographic accessibility than urban areas.

**Conclusions:**

During COVID-19 vaccine scarcity or abundance, deprived and ethnically concentrated urban areas had similar or higher access compared to less deprived areas. However, rural deprived areas experienced lower geographic accessibility. Access to pharmacies can be enhanced in rural deprived areas by incentivization and outreach. Further research examining whether this geographic accessibility variance influenced vaccine uptake and infection rates.

**Supplementary Information:**

The online version contains supplementary material available at 10.1186/s12889-025-24929-w.

## Introduction

Coronavirus disease 2019 (COVID-19) is an infectious disease of major public health burden globally and was first detected in Canada in February 2020 [1]. COVID-19 vaccination has been shown to prevent severe complications and hospitalization and reduce mortality [2]. In Canada, community pharmacists offered access to vaccination [3, 4], including through convenient locations, often with walk-in availability and extended operating hours [5, 6]. Pharmacists are the most accessible health care professionals and most Canadians live within walking distance of a community pharmacy [7, 8]. This convenience has been cited by the Canadian public as a key vaccination facilitator, with pharmacies being the most common setting for influenza vaccination [9]. On March 10, 2021, participating pharmacies across Ontario began offering COVID-19 vaccination as part of the mass immunization campaign [10]. 

In the early years of the pandemic, economically disadvantaged and ethnic minority populations faced higher risks of both infection and mortality from COVID-19 [11, 12]. Ontario’s COVID-19 vaccine rollout went through several phases—including scarcity, abundance, and a return to normalcy. Throughout each phase, the issue of inequity remained critical, as decisions related to vaccine distribution were essential to ensuring that those most vulnerable to adverse outcomes received the preventive care they needed. Inequity also manifested in terms of access to preventative measures such as less ability to physically distance [13] or difficulty obtaining a vaccination [14]. For example, in 2021, vaccine uptake and intent were found to be negatively associated with level of education, while vaccination intent was higher among those identifying as belonging to a visible minority [15]. Additionally, vaccination coverage among individuals in Ontario experiencing homelessness was significantly lower than the general population (25% vs. 86%) [16]. 

Questions have emerged about inequitable access to COVID-19 vaccinations, where those who need vaccines the most do not get them, or do not get them at the right time [11]. The inverse care law [17] – that “the availability of good medical care tends to vary inversely with the need for it in the population served” [18] – becomes important in this context [19], especially when resources are scarce [20]. An investigative media analysis by the Canadian Broadcasting Corporation (CBC) in April 2021 found that only 19% of pharmacies located in the 10 neighborhoods in Toronto with the highest COVID-19 infection rates offered COVID-19 vaccination, compared to 43% of pharmacies in the 10 neighborhoods with the lowest COVD-19 infection rates [21]. Most studies on geographic accessibility examined either a single point in time [22] or broad intervals (e.g., one to two years) during the pandemic [23], limiting their ability to capture granular shifts in vaccine accessibility and distribution across population groups as the situation evolved.

Even before the pandemic, this inverse relationship was observed related to general pharmacy practice [24], where, for example, higher income communities had better access to expanded pharmacist prescribing services [25]. Research in the United States also identified a shortage of community pharmacies in racially-marginalized and low-income communities [26, 27], with other work finding lower pharmacist vaccinator availability in Ontario in rural communities [28]. However, little is known about the relationship between access to COVID-19 vaccine in pharmacies and community characteristics including socioeconomic status and ethnic diversity.

Ensuring that individuals who need vaccines the most can readily access them is important to successfully implement equitable immunization campaigns. This is consistent with the PROGRESS-Plus [29] and the WHO Commission frameworks [30] (both of which identifies place of residence and distribution of resources as a major determinant of health). As such, the objective of this study is to examine if there was inequality in the geographic accessibility of community pharmacies offering COVID-19 vaccination in Ontario relative to rurality, material deprivation, and ethnic concentration during the first nine months of service availability. This study builds on previous research efforts of this relationship [22, 23], by employing a repeated cross-sectional design to characterize evolving patterns of pharmacy-based vaccine accessibility across multiple phases of the pandemic.

## Methods

### Study design and setting

This was a repeated cross-sectional observational geospatial study which examined community pharmacy geographic accessibility offering COVID-19 vaccination by Dissemination Area (DA) in Ontario, stratified by the material deprivation index and ethnicity concentration index. A DA is defined as a small, relatively stable geographic unit comprising one or more adjacent dissemination blocks with a target population of 400–700 individuals [15]. DAs were used to compute geographic accessibility, as they are the smallest geographic polygons that can be used to depicting where people live, and thus, from where they travel to access pharmacy services.

### Data source

A list of all pharmacies offering COVID-19 vaccination was obtained from the Ontario Ministry of Health’s publicly available website [31]. Data extraction from this website was performed using Python^®^ 2.7.8 and the BeautifulSoup library^®^ software outside of business hours (before 9 am or after 5 pm). A shape file containing DA boundaries, characteristics and population estimations was obtained from Statistics Canada’s 2016 census of the population [32, 33]. Marginalization 2016 indices at the DA level were obtained from the Public Health Ontario website [34, 35], and were used to classify each area into one of five quintiles of vulnerability. Each DA was assigned to a quintile of the material deprivation, and ethnic concentration indices in this study, with quintile 1 being the least deprived and quintile 5 being the most deprived.

Rural DAs were identified using the Metropolitan Influenced Zone (MIZ) classification system [36]. DAs with a degree of influence of moderate, weak, or no influence were considered rural. Specifically, “moderate” influence corresponds to 5–30% of the population commuting to an urban core, “weak” influence refers to less than 5% commuting, and “no influence” captures areas with no residents commuting to a metropolitan region [36]. 

The geospatial location of community pharmacies was obtained from Environics Analytics Business Data for Ontario [37], while the road network, which is used to calculate the driving lengths needed to reach a pharmacy, was obtained from DMTI CanMap streetfiles [38]. 

### Dates of analysis

The accessibility index was measured on eight selected dates, determined by reviewing previous literature [15, 21, 39] on this topic and policy documents from the Ontario Pharmacists Association to reflect dates when major changes in COVID-19 vaccine availability and eligibility criteria were reported. Selection also considered dates when limited or large supplies of vaccines were released into the community so that their distribution could be analyzed according to the accessibility score (described below). Eight specific dates were selected to reflect key phases of Ontario’s COVID-19 vaccine rollout and to assess shifts in pharmacy accessibility across distinct policy milestones. Between late April and May, eligibility expanded rapidly—from individuals aged 45 + in hot spot areas to all youth aged 12+. Starting in November, booster eligibility widened in phases: first to high-risk groups, then to those 50+, and finally all adults by December 20. The dates used in the analysis, along with their rationale, are listed in Appendix 1.

### Data analysis

Analysis was conducted using SAS^®^ 9.4 and ESRI ArcGIS^®^ 10.5.1 software. The geospatial locations of pharmacies for analysis were obtained by initially linking pharmacy names from the downloaded records to the corresponding business names and addresses in the 2021 Environics business database obtained from the geospatial center at the University of Waterloo. For pharmacies not linked to the Environics business dataset, geospatial locations were determined using postal code geographic coordinates, utilizing the Postal Code Conversion File Plus (PCCF+) version 6 A which matches the postal code geography with the Census geography. Pharmacies without geospatial coordinates were excluded from the analysis due to presumed address inaccuracies that prevented reliable mapping and assignment to geographic units.

After converting the records of pharmacies to geospatial locations (i.e., coordinates), service areas for each pharmacy and geographic accessibility indices for each DA were determined. Then, we calculated the average geographic accessibility to pharmacies offering COVID-19 vaccination among all DAs in each of the five quintiles (of material deprivation and ethnic concentration).

**Fig. 1:**
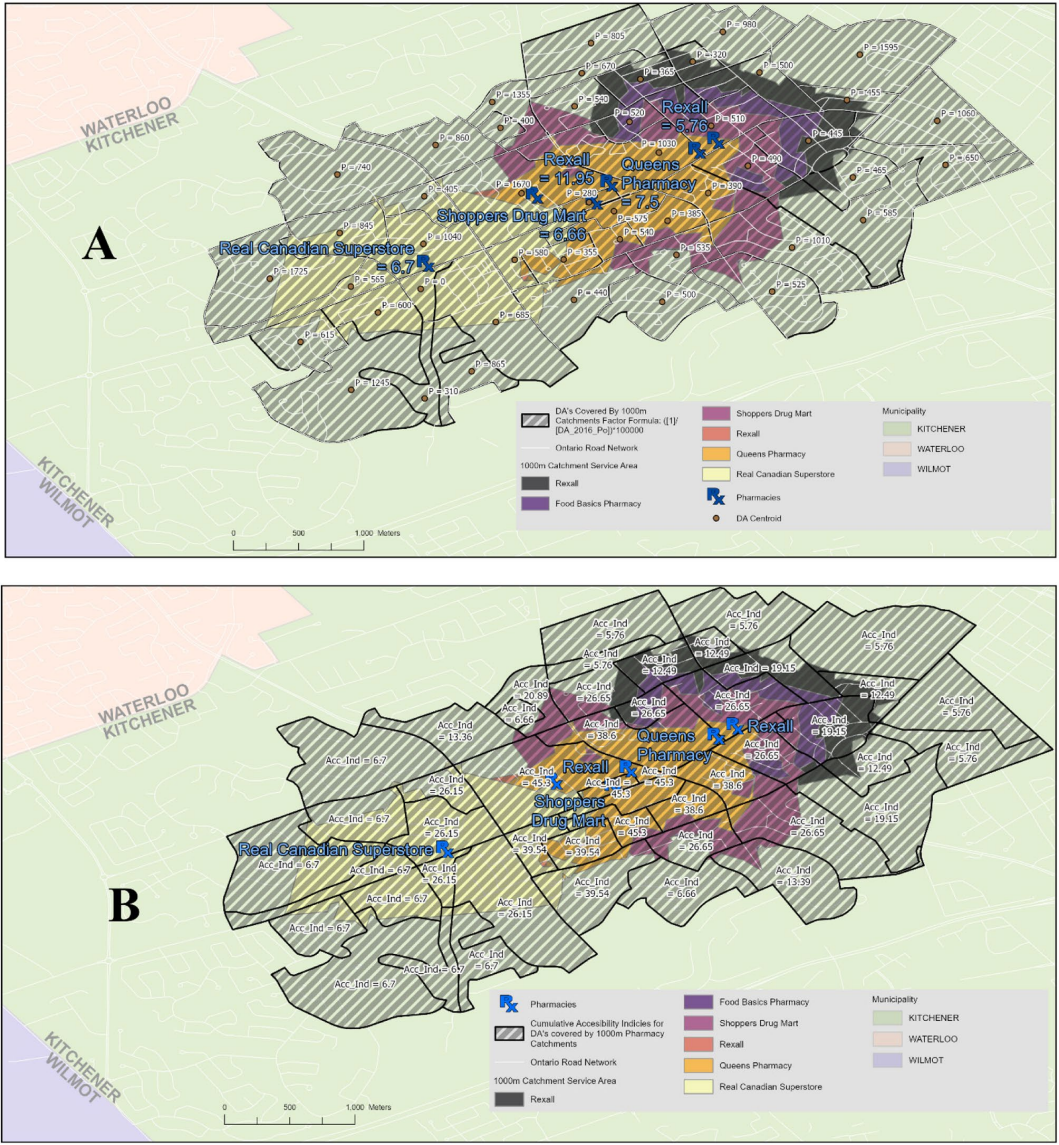
Computing accessibility to pharmacies offering COVID-19 vaccination in the city of Waterloo The map is segmented by DisseminationAreas (DAs). White lines denote road networks. Pharmacy locations are marked with theirnames and corresponding pharmacy-to-population ratios. Catchment areas within 1,000 metersare visualized using shaded regions. The population of each DA is labeled (A), while theaccessibility score—defined as the sum of all pharmacy-to-population ratios for pharmacieswhose catchment areas intersect with the DA boundaries—is also displayed (B)

The accessibility index was calculated using the two-step floating catchment area (2SFCA) method [40]. We defined the service (i.e., catchment) area for each pharmacy as a 1,000 m, 1,500 m, and 3,000 m driving distance in urban areas and a 10,000 m in rural areas. For each pharmacy where vaccine was available, we summed the population of all DAs whose centroid fell within the service area. We then calculated the pharmacy-to-population ratio by dividing the number of pharmacies (i.e., 1) by the total population residing in that area. Given how small these values can be (e.g., 1 ÷ 1,500 = 0.000667), scaling by a factor of 100,000 was employed to facilitate visualization and analysis. Then, we computed the accessibility score of each DA by summing the pharmacy-to-population ratios of all pharmacies with service areas that intersect with the DA boundary. A higher accessibility index score indicates that it is easier for persons living in each DA to access pharmacies offering COVID-19 vaccination, while a lower score indicates more difficulty accessing a vaccine. Figure 1 illustrates an example of computing accessibility to pharmacies offering COVID-19 vaccination in the city of Waterloo using the 2SFCA method. All DAs, including those with low population counts, were included in the analysis.

We depicted the trend of this geographic accessibility at driving distances of 1,000, 1,500, and 3,000 m in urban areas and 10,000 m in rural areas. The selection of driving distances for the catchment area size was based on previous studies within the Canadian context [41]. While there is no universally agreed-upon catchment area size, using variable catchment are is recommended particularly in rural areas where services are more dispersed [42]. This is because smaller catchment areas can introduce measurement uncertainties, leading to model instability [43]. Conversely, although larger catchment sizes tend to provide more stable models, they may dilute the differences in acceptability between areas [44]. Subsequently, the average accessibility to pharmacies among all DAs in each quintile was compared across the five quintiles using one-way Analysis of Variance (ANOVA). We assessed ANOVA assumptions using Levene’s test for variance for homogeneity and Q–Q plots for residual normality. When assumptions were violated, Welch’s ANOVA was used with p-values and Eta Squared (η²) reported to convey the statistical and practical significance. Significant was set at *p* < 0.05; η² >0.06 indicated medium effects, and η²≥ 0.14 indicated large effects [45]. 

Ethics approval for this study was not required as the data used is publicly available [46]. 

## Results

Of 20,161 DAs in Ontario, 18,701 were categorized as urban, while 1,458 were categorized as rural and 2 could not be determined. A total of 15,174 unique pharmacy records were extracted to complete the analysis over the study period. The average linkage rate to geographic coordinates from the Environics business database or PCCF + was 92.9% (i.e., about 7.1% of pharmacies were excluded from the analysis) and ranged from 91.3% to 94.4%. Appendix 2 shows the flow of the extracted records of our study. Three distinct eras in service availability were identified over the study period: [1] Intermediate (April 27th, May 10, and May 23ed, 2021), during which approximately 1,400 pharmacies in Ontario were providing COVID-19 vaccination; [2] Scarcity (May 12, 2021), when only 135 pharmacies were offering the service; and [3] Abundance (November 5th to December,20th 2021), with more than 2,700 pharmacies offering the service.

### Urban areas

**Fig. 2:**
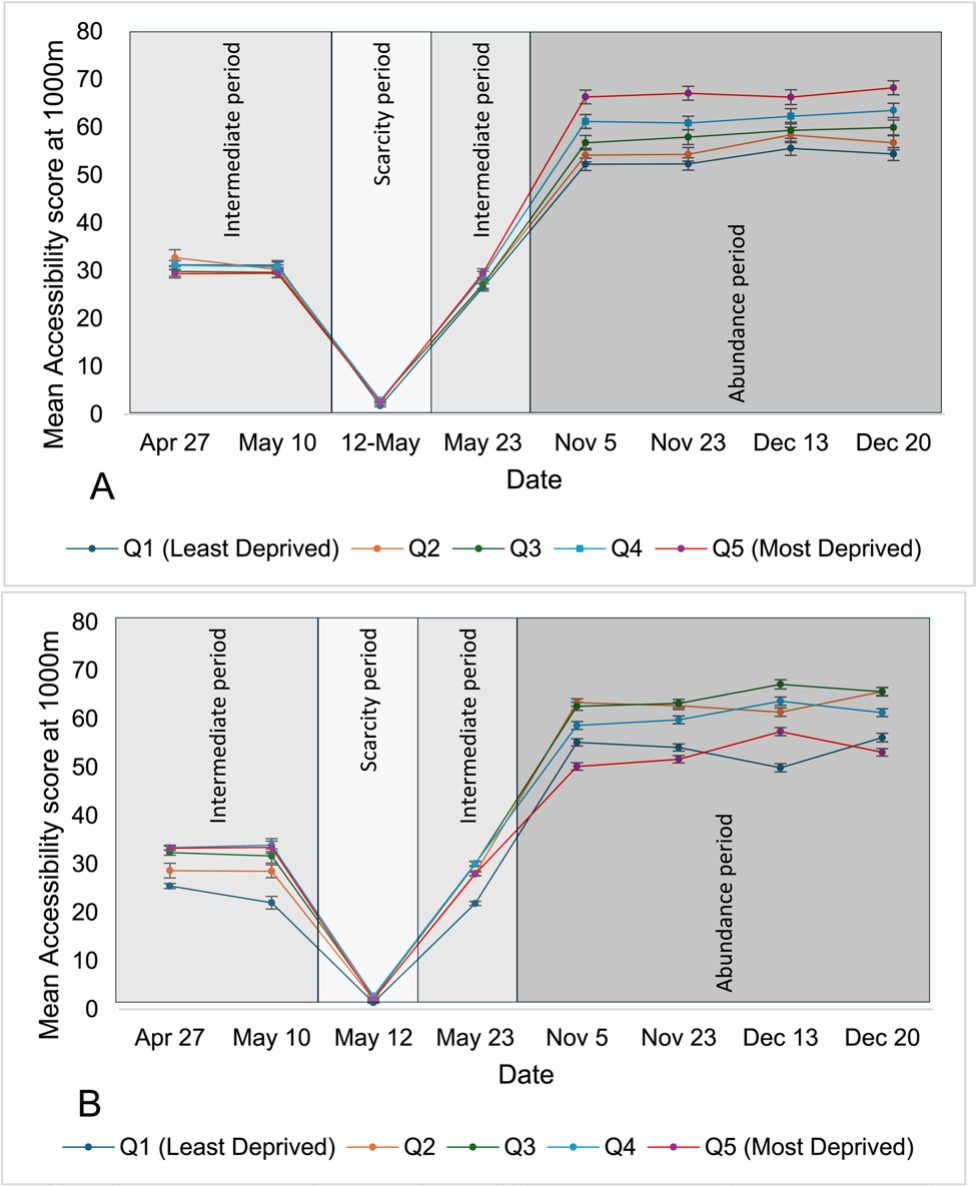
Mean accessibility at 1000m of pharmacies offering COVID-19 vaccination across Ontario’s urban areas, stratified by material deprivation (A) and ethnic concentration (B) quintiles over the study period (Accessibility Index is calculated by summing scaled pharmacy-to-population ratios (1 ÷ total DA population × 100,000) for all pharmacies whose catchment areas intersect with each DA; scores are then categorized into quintiles for comparative analysis). (Accessibility Index is calculated by summing scaled pharmacy-to-population ratios (1 ÷ total DA population × 100,000) for all pharmacies whose catchment areas intersect with each DA; scores are then categorized into quintiles for comparative analysis)

**Fig. 3:**
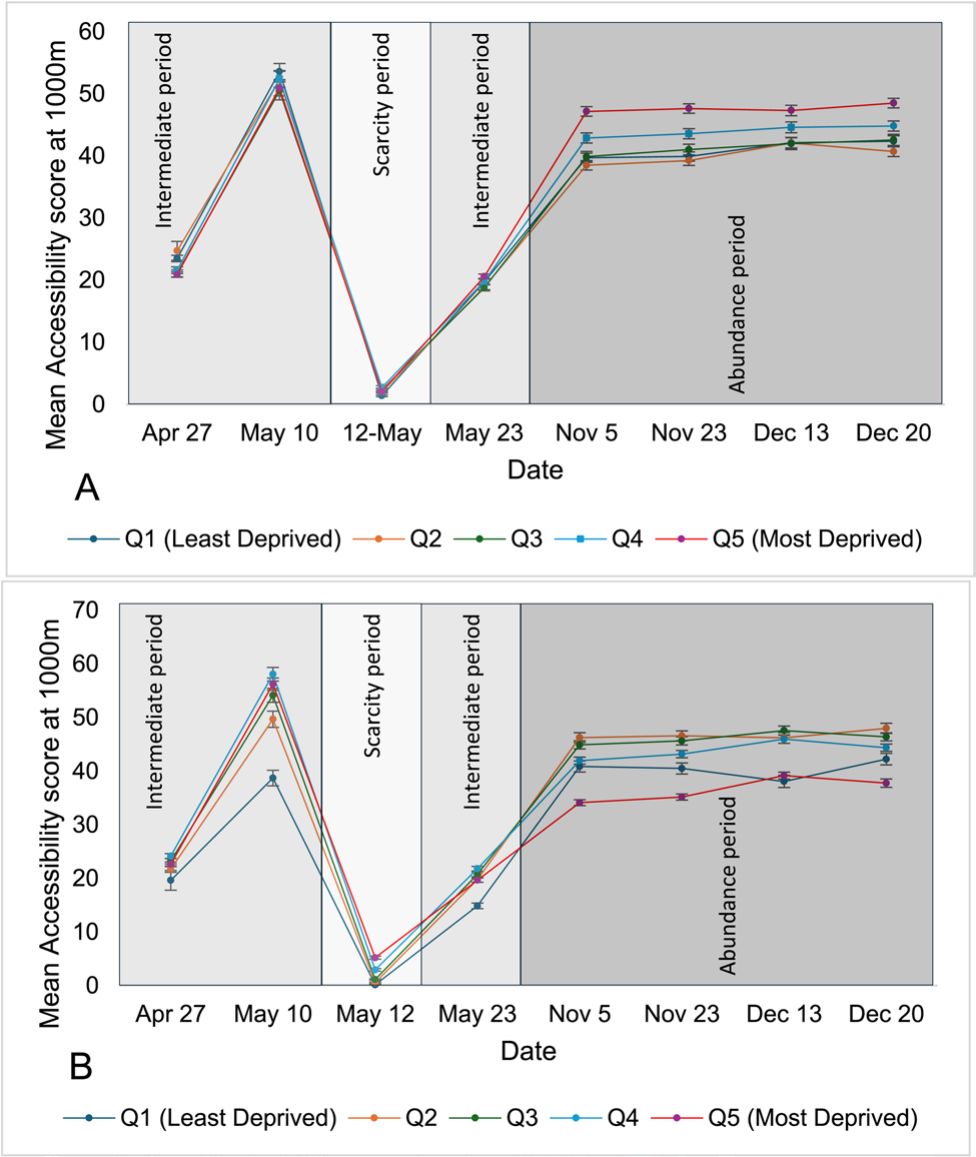
Mean accessibility at 1,500m of pharmacies offering COVID-19 vaccination across Ontario’s urban areas, stratified by material deprivation (**A**) and ethnic concentration (**B**) quintiles over the study period (Accessibility Index is calculated by summing scaled pharmacy-to-population ratios (1 ÷ total DA population × 100,000) for all pharmacies whose catchment areas intersect with each DA; scores are then categorized into quintiles for comparative analysis). (Accessibility Index is calculated by summing scaled pharmacy-to-population ratios (1 ÷ total DA population × 100,000) for all pharmacies whose catchment areas intersect with each DA; scores are then categorized into quintiles for comparative analysis)

Overall, in urban areas, geographic accessibility improved (as seen by an increasing accessibility index) as the driving distance from pharmacies was extended from 1,000 to 1,500 and 3,000 m (Fig. 2), respectively. Accessibility improved over the intermediate availability era and was significantly reduced during the scarcity period and then increased and remained stable during periods of abundance. Of note, there was no geographic accessibility (i.e., accessibility index of 0) during the scarcity period in rural areas, when all pharmacies providing COVID-19 vaccination were more than 10,000 m away from all rural DAs (Fig. 3).

**Fig. 4:**
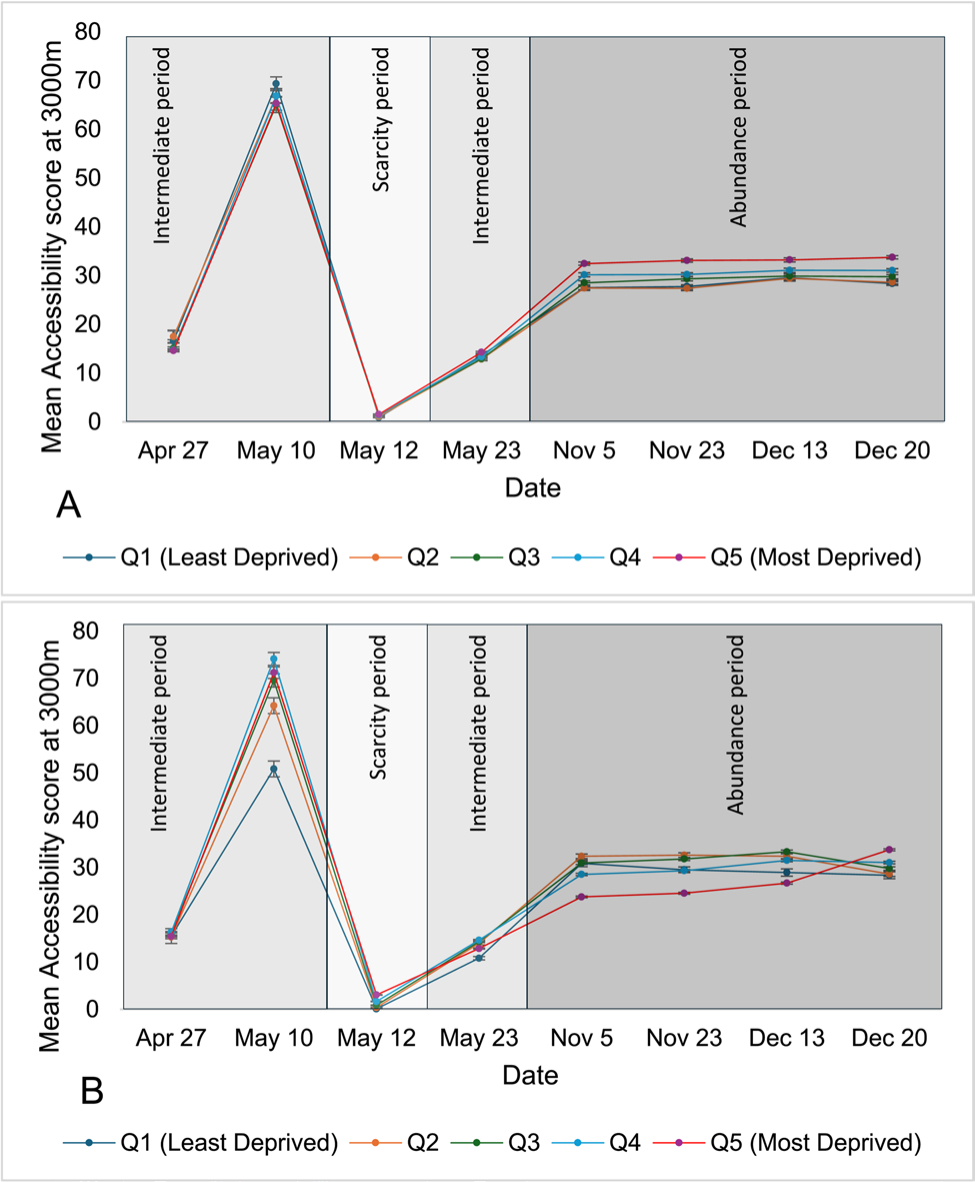
Mean accessibility at 3,000m of pharmacies offering COVID-19 vaccination across Ontario’s urban areas, stratified by material deprivation (**A**) and ethnic concentration (**B**) quintiles over the study period. (Accessibility Index is calculated by summing scaled pharmacy-to-population ratios (1 ÷ total DA population × 100,000) for all pharmacies whose catchment areas intersect with each DA; scores are then categorized into quintiles for comparative analysis)

**Fig. 5:**
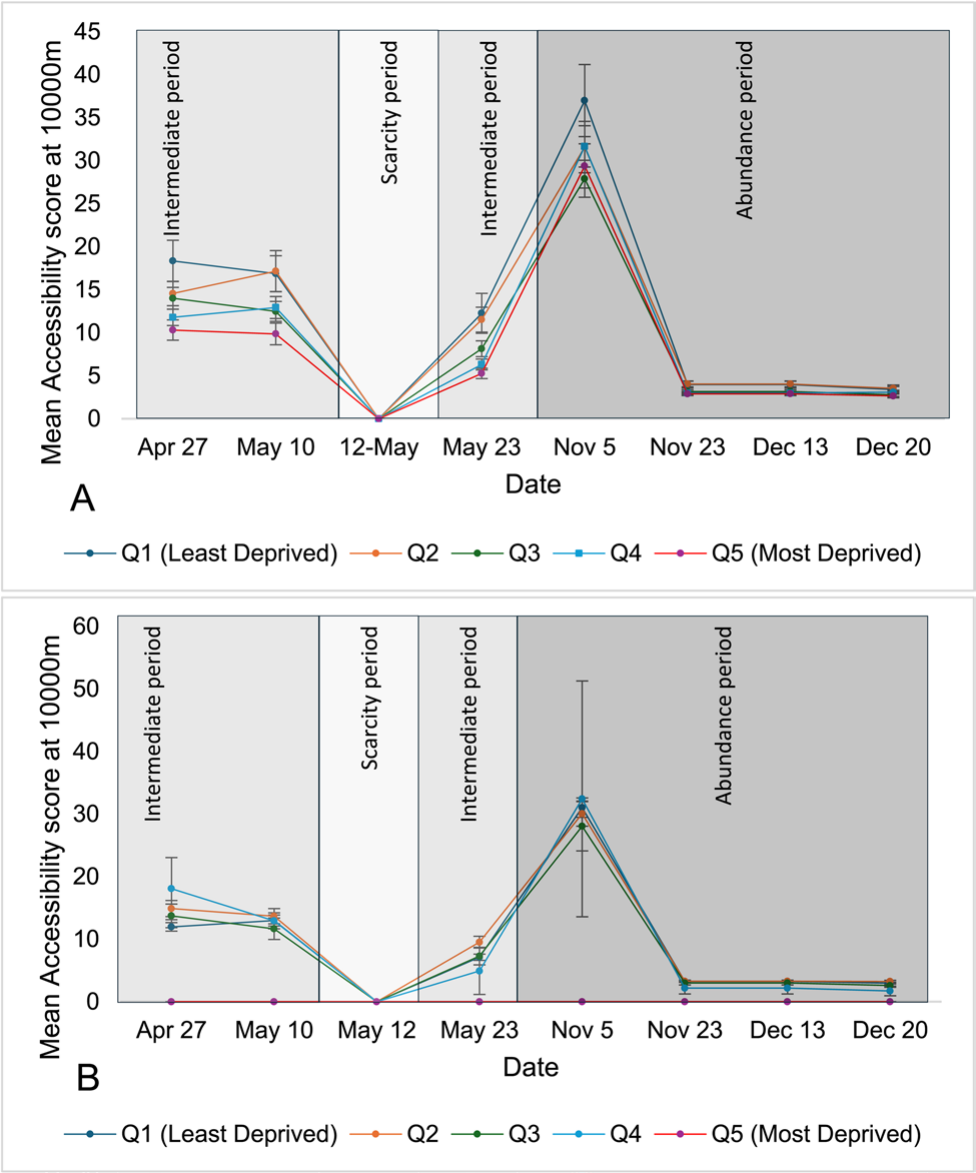
Mean accessibility at 10,000m of pharmacies offering COVID-19 vaccination across Ontario’s rural areas, stratified by material deprivation (**A**) and ethnic concentration (**B**) quintiles over the study period. (Accessibility Index is calculated by summing scaled pharmacy-to-population ratios (1 ÷ total DA population × 100,000) for all pharmacies whose catchment areas intersect with each DA; scores are then categorized into quintiles for comparative analysis)

Geographic accessibility scores by quintile of material deprivation or ethnic concentration in urban and rural areas are shown in Tables 1 and 2, respectively and the trend of these accessibility scores are shown in Figs. 2, 3, 4 and 5. In urban areas over most studied dates, when the service area was measured at 1,000 m, accessibility to pharmacies offering COVID-19 vaccine was significantly higher in DAs with greater material deprivation and/or ethnic concentration, except early in the vaccination campaign (April 27 and May 10 dates) when there was no difference. This higher availability was observed during the scarcity period when accessibility was 1.76 and 0.18 in the least materially deprived and least ethnically concentrated DAs, respectively, versus 2.52 and 6.55 in the most materially deprived and most ethnically concentrated DAs.

**Table 1:** Mean accessibility of pharmacies offering COVID-19 vaccination across ontario’s urban dissemination areas stratified based on quintiles of material deprivation and ethnic concentration over the study period

DA Deprivation Quintile	Material deprivation			Ethnic concentration			Material deprivation			Ethnic concentration			Material deprivation			Ethnic concentration		
	1000-meter driving distance						1500-meter driving distance						3000-meter driving distance					
	April 27th																	
	Accessibility score	Welch’s ANOVA *p*-value	Eta-Square	Accessibility score	Welch’s ANOVA *p*-value	Eta-Square	Accessibility score	Welch’s ANOVA *p*-value	Eta-Square	Accessibility score	Welch’s ANOVA *p*-value	Eta-Square	Accessibility score	Welch’s ANOVA *p*-value	Eta-Square	Accessibility score	Welch’s ANOVA *p*-value	Eta-Square
1 (least deprived)	31.22	0.558	0.0002	25.46	< 0.0001	0.0047	23.49	0.002	0.0009	19.62	< 0.0001	0.0061	16.55	< 0.0001	0.0018	15.5	< 0.0001	0.0046
2	32.72			28.65			24.72			21.72			17.47			15.28		
3	29.86			32.38			21.10*			23.22			15.09			15.96		
4	31.17			33.39			21.60†			24.15			14.73			16.32		
5 (most deprived)	29.39			33.27			20.9			22.63			14.59†			15.42		
*May 10th*																		
1 (least deprived)	31.18	0.66	0.0001	22.02	< 0.0001	0.0051	53.58	0.489	0.0002	38.71	< 0.0001	0.0065	69.33	0.2329	0.0003	50.85	< 0.0001	0.0076
2	30.27			28.52			52.28			49.68			66.84			64.23		
3	29.66			31.67			50.47			54.14			65.01			69.57		
4	30.85			33.91			52.41			58.07			66.82			74.12		
5 (most deprived)	29.46			33.48			50.93			56.23			65.32			71.21		
*May 12th*																		
1 (least deprived)	1.76	0.0001	0.0013	0.18	< 0.001	0.0326	1.39	< 0.0001	0.0014	0.15	< 0.0001	0.0242	0.92	< 0.0001	0.004	0.11	< 0.0001	0.0854
2	2.96			0.74			2.01			0.54			1.12			0.38		
3	2.93			1.49			2.35			1.13			1.38			0.86		
4	2.9			3.4			2.74			2.93			1.45			1.67		
5 (most deprived)	2.52			6.55			2.06			5.2			1.55			3.05		
*May 23rd*																		
1 (least deprived)	26.55	0.0707	0.0004	21.87	< 0.0001	0.0027	19.73	0.0241	0.0006	14.87	< 0.0001	0.0066	13.66	< 0.0001	0.0014	10.81	< 0.0001	0.0088
2	27.23			28.09			18.93			19.77			12.85			13.95		
3	27.09			30			18.76			20.74			12.92			14.29		
4	28.99			30.12			19.82			21.8			13.34			14.64		
5 (most deprived)	29.52			27.94			20.58			19.66			14.28			12.86		
*Nov 5th*																		
1 (least deprived)	52.25	< 0.0001	0.0034	55.15	< 0.0001	0.0033	39.71	< 0.0001	0.0042	40.86	< 0.0001	0.0081	27.51	< 0.0001	0.006	30.87	< 0.0001	0.0158
2	54.15			63.38			38.49			46.25			27.45			32.37		
3	56.75			62.6			39.89			44.9			28.52			30.96		
4	61.2			58.64			42.89			41.96			30.16			28.53		
5 (most deprived)	66.33			50.18			47.16			34.12			32.46			23.78		
*Nov 23rd*																		
1 (least deprived)	52.33	< 0.0001	0.0036	54.1	< 0.0001	0.0029	39.91	< 0.0001	0.004	40.5	< 0.0001	0.0075	27.76	< 0.0001	0.0078	29.52	< 0.0001	0.0153
2	54.33			62.72			39.25			46.59			27.37			32.62		
3	57.93			63.19			41.02			45.64			29.33			31.81		
4	60.87			59.8			43.57			43.18			30.22			29.32		
5 (most deprived)	67.1			51.66			47.63			35.17			33.09			24.56		
*Dec 13th*																		
1 (least deprived)	55.58	< 0.0001	0.0014	49.91	< 0.0001	0.0035	42.13	< 0.0001	0.0015	38.08	< 0.0001	0.0052	29.56	< 0.0001	0.0027	28.94	< 0.0001	0.0082
2	58.39			61.4			42.07			46.2			29.42			32.39		
3	59.35			67.14			41.97			47.56			29.89			33.33		
4	62.28			63.66			44.6			45.97			31.08			31.5		
5 (most deprived)	66.28			57.37			47.31			39.17			33.21			26.7		
*Dec 20th*																		
1 (least deprived)	54.4	< 0.0001	0.003	56.14	< 0.0001	0.0032	42.36	< 0.0001	0.0027	42.24	< 0.0001	0.005	28.36	< 0.0001	0.0071	30.93	< 0.0001	0.0127
2	56.74			65.65			40.71			47.99			28.61			32.93		
3	59.94			65.63			42.58			46.39			29.78			32.24		
4	63.52			61.29			44.81			44.37			31.05			30		
5 (most deprived)	68.24			53.09			48.5			37.76			33.75			25.59		

**Table 2:** Mean accessibility within a 10,000-meter driving distance to pharmacies offering COVID-19 vaccination across ontario’s rural dissemination areas stratified based on quintiles of material deprivation and ethnic concentration over the study period

	Material deprivation			Ethnic concentration		
April 27th						
*DA Deprivation Quintile*	Accessibility	Welch’s ANOVA p-value	Eta-Square	Accessibility	Welch’s ANOVA p-value	Eta-Square
*1 (least deprived)*	18.35	0.011	0.0123	11.96	0.1616	0.0046
*2*	14.56			14.89		
*3*	14			13.72		
*4*	11.79			18.08		
*5 (most deprived)*	10.31			-		
*May 10th*						
*1 (least deprived)*	16.87	0.0143	0.0108	12.98	0.066	0.0014
*2*	17.16			13.67		
*3*	12.48			11.66		
*4*	12.93			12.93		
*5 (most deprived)*	9.86			-		
*May 12th*						
*1 (least deprived)*	-	-	-	-	-	-
*2*	-	-		-	-	
*3*	-	-		-	-	
*4*	-	-		-	-	
*5 (most deprived)*	-	-		-	-	
*May 23rd*						
*1 (least deprived)*	12.26	< 0.0001	0.0274	7.11	0.1525	0.005
*2*	11.52			9.52		
*3*	8.14			7.29		
*4*	6.31			4.91		
*5 (most deprived)*	5.27			-		
*Nov 5th*						
*1 (least deprived)*	36.99	0.357	0.003	31.03	0.9092	0.0003
*2*	31.58			30.06		
*3*	27.9			28.05		
*4*	31.66			32.43		
*5 (most deprived)*	29.39			-		
*Nov 23rd*						
*1 (least deprived)*	3.98	0.0097	0.0139	3.25	0.5988	0.0011
*2*	4.04			3.27		
*3*	3.16			2.99		
*4*	2.99			2.17		
*5 (most deprived)*	2.9			-		
*Dec 13th*						
*1 (least deprived)*	3.98	0.0097	0.0139	3.25	0.5988	0.0011
*2*	4.04			3.27		
*3*	3.16			2.99		
*4*	2.99			2.17		
*5 (most deprived)*	2.90			-		
*Dec 20th*						
*1 (least deprived)*	3.41	0.133	0.006	2.98	0.1147	0.0023
*2*	3.53			3.23		
*3*	2.78			2.59		
*4*	3.09			1.72		
*5 (most deprived)*	2.65			-		

Findings were largely similar to those found at a service area of 1,500 m. However, accessibility at 1,500 m was lower for DAs with the highest material deprivation on April 27, and DAs with the highest ethnic concentration later in the study period on November 5 and December 20.

At a service area of 3,000 m, accessibility to pharmacies offering COVID-19 vaccine was significantly higher in the least materially deprived DAs on April 27, versus December 20 in the DAs with lowest ethnic concentration. The same trend of improved accessibility during the scarcity period for DAs with greatest material deprivation and lowest ethnic concentration was observed at this service area.

Although statistical significance was observed, the overall effect size remained small, as reflected by consistently low eta-squared (η²) values at 1,000, 1,500 and 3,000 m. However, during the time of scarcity the effect size was medium for 3,000-meter driving distance when DAs were categorized by ethnic concentration quintile with a value of 0.0854 (Table 1).

### Rural areas

As there was no accessibility to pharmacies offering COVID-19 vaccinations in rural areas during the scarcity period, a comparison across deprivation quintiles was not possible. However, during intermediate and abundance periods, the most deprived areas exhibited generally lower accessibility when measured by material deprivation and demonstrated similar accessibility when measured by ethnic concentration (Table 2). The effect size of this difference was also small.

## Discussion

We examined the geographic accessibility of Ontario community pharmacies offering COVID-19 vaccination in urban and rural areas considering the community-level material deprivation and ethnic concentration. While geographic accessibility changed over time, overall, materially deprived and ethnically concentrated urban areas demonstrated either better or similar geographic accessibility to pharmacies offering COVID-19 vaccine compared to less deprived and less ethnically concentrated areas. During vaccine shortages, more deprived areas exhibited higher geographic accessibility than their less deprived counterparts. However, in rural areas, there was little to no geographic accessibility during supply shortages. Conversely, when there was an intermediate or abundant supply, deprived areas generally had lower geographic accessibility to pharmacies offering the COVID-19 vaccine.

Despite its importance [47, 48], few studies have specifically assessed geographical accessibility of COVID-19 vaccination in community pharmacies for vulnerable populations [49, 50]. Our findings suggest that Ontario generally provided equitable opportunities for populations living in more materially deprived or ethnically concentrated areas to access COVID-19 vaccination through community pharmacies in 2021- at least from a geographic availability point of view. This contrasts with vaccine deserts in the US (areas with limited access to vaccination sites including community pharmacies) which were found to be predominantly concentrated in rural areas and among other high-need populations such as people of color and older adults [51]. This may reflect the different approaches followed by the Government of Ontario to promote vaccine equity, including community-led efforts [52] and tailored approaches for marginalized groups such as immigrants, rural residents, and people with disabilities [53]. In fact, during times of shortage, more vaccine supply was allocated to areas with higher material deprivation or ethnic concentration. This is in contrast to access to healthcare resources which was found to have inequitable geographic accessibility during the COVID-19 pandemic [54–56]. 

Our results showed that the vaccine was readily available in Ontario to vulnerable populations who needed it the most, such as people living in materially deprived and ethnically highly concentrated areas. However, that does not necessarily mean that vaccine uptake was optimal in those areas, as our data shows that pharmacies offering COVID-19 vaccines were available in those areas, but uptake may have been low. Vaccine uptake can be influenced by behavioral factors such as vaccine hesitancy and lack of trust in healthcare systems or government initiatives. Social determinants, such as affordability, cultural beliefs and historical contexts, can also play significant roles. Addressing these aspects is essential for developing targeted interventions that promote vaccine acceptance.

While this study indicates that access to COVID-19 vaccination through community pharmacies in 2021 was generally equitable in urban areas, rural areas had no geographic accessibility to pharmacies offering COVID-19 vaccination within a 10-kilometer driving distance during supply shortages. Additionally, when there is sufficient supply, rural areas with higher material deprivation exhibited lower geographic accessibility to pharmacies offering vaccination compared to more affluent areas. This reflects the ongoing gap in equity for Canadians living in rural areas despite some recent progress [57]. People who live in rural areas were found to be at a higher risk of COVID-19 infection hospitalization and mortality, contrary to the assumption of a natural social distancing lifestyle [58, 59]. Several policy solutions can be considered to address the observed gaps in rural pharmacy geographic accessibility. Mobile vaccination units may offer a practical means of reaching communities without fixed-site access, particularly in sparsely populated regions [60]. Similarly, temporary rural clinics could be deployed during periods of high demand to supplement existing infrastructure [61]. These strategies, may provide evidence-based geographically responsive approach to future vaccine rollouts.

Although recent studies have questioned the notion that rural areas are more vulnerable to COVID-19 infections [62], at least in most Canadian provinces in terms of mortality and acute illness [62], our study has shown that Canadians living in rural areas had limited access to pharmacies offering COVID-19 vaccines. In fact, during times of vaccine scarcity, Ontarians living in rural areas had no access to any such pharmacies. Approximately 17% of Ontario’s population live in rural areas—that’s about 2.5 million people out of 14 million [63]. Our findings may partially explain the lower vaccination coverage in these areas [14, 53, 64]. While vaccine hesitancy may be more prevalent in rural areas [65], our results showed that the impact of geographically accessible vaccination services cannot be underestimated. Adequate and universal distribution of vaccination-offering pharmacies is essential to ensure that vaccination campaigns can successfully reach all residents including rural dwelling individuals.

Both material and social attributes can impact healthcare system use and health outcomes [66, 67]. Material factors include income, education, and employment, while social factors encompass the degree of supportive social networks or experienced social isolation as well as ethnicity. Accessibility should be considered in light of these factors. Our results indicate that the trend observed when measuring geographic accessibility across material deprivation levels does not always align with the trend observed across ethnic concentration levels. These results suggest that factors contributing to health service use and outcomes can be considered intersectional, where multiple factors impact individuals’ health simultaneously. Public health programs should consider several indices of communities’ material and social attributes when allocating vaccines to accessible pharmacies. In addition to ensuring geographic accessibility and connection to care providers [68], other research has highlighted that vaccination campaigns can benefit from targeted media advertisements (including local and social media); [69] technology to help users locate vaccination services easily with real-time data; [70] engaged health facilities and local stewards, champions, and representatives to encourage vaccination, and; explicitly guide community members to accessible providers of vaccinations [71, 72]. 

### Limitations

Our study used comprehensive publicly available data across the entire province of Ontario over eight dates to capture variations in access during different periods of vaccine availability. The mean linkage success of the data was very high; however, several limitations should be considered. First, we assumed that people were serviced by the pharmacy closest to their residence; however, individuals can travel and be serviced by whichever pharmacy is convenient for them, such as one located near their workplace. Second, data related to material deprivation are based on a set number of parameters, such as median income and single mothers, and is not individualized. In addition, using DA-level indices may not accurately reflect individual experiences within each DA. This can lead to ecological fallacy, where the concordance with individuals’ personal vulnerability is not always perfect [73]. On the other hand, we used Statistics Canada’s 2016 Census of Population and Marginalization Indices to estimate areas’ populations and vulnerabilities, as this study was conducted before the release of 2021 estimates. Nevertheless, population growth is expected to be similar across areas, and a significant shift in vulnerability quintiles is not anticipated. Third, we assumed that if a pharmacy was listed on the website as offering COVID-19 vaccination, there was vaccine stock available for their use. However, we acknowledge that even if a pharmacy offered the service, product availability was widely variable over the study period. This study did not account for whether the reported pharmacies from the database had vaccine doses available consistently or not. The level of stock supply was not publicly available, and detailed distribution data are needed to assess if pharmacies’ supply varied by the communities they serve. Lastly, our pharmacy dataset achieved approximately 93% geocoding linkage, supporting a robust spatial analysis across DAs. While pharmacies with missing location data were excluded from the analysis, we consider systematic missingness to be unlikely, as there’s no clear indication that geocoding success was influenced by pharmacy location relative to material deprivation or ethnic concentration quintiles. Nonetheless, it remains possible that some pharmacy-level characteristics linked to geographic context—such as service type, hours of operation, or pharmacy size—may have contributed to patterns of missingness, introducing potential bias in geographic accessibility estimation, especially in regions where the population is sparsely distributed.

## Conclusion

Our findings suggest that there was either higher or similar geographic accessibility to pharmacies offering COVID-19 vaccination among quintiles of material deprivation and ethnic concentration in urban areas of Ontario in 2021; however, rural areas experienced no or limited access during supply shortages. When supply was available, materially deprived rural areas had lower geographic accessibility compared to affluent areas. The findings in urban areas are promising as they go against the inverse care law. In contrast, improvements for geographic accessibility in rural areas can still be made in future vaccine rollout programs. As the risks of COVID-19 and benefits of additional vaccine doses remain ongoing, it is imperative that those at greater risk of contracting COVID-19 or experiencing severe illness have access to vaccination. To support equitable vaccine site placement in future rollouts, planning efforts may benefit from the integration of spatial accessibility data into real-time operational tools that can identify underserved areas and guide timely resource deployment. Future research should investigate whether pharmacies geographic accessibility is correlated to vaccination uptake and COVID-19 infection rates.

## Supplementary Information

Supplementary Material 1. Appendix 1: Dates of analysis and their respective rationale.

Supplementary Material 2. Appendix 2: Flowchart of pharmacy records into the study.

## Data Availability

The dataset analyzed during the current study was obtained from publicly available online sources. The compiled database is available from the corresponding author upon reasonable request.

## Abbreviations

ANOVA: Analysis of Variance
CBC: Canadian Broadcasting Corporation
COVID-19: Coronavirus Disease of 2019
DMTI: Digital Map Products
DA: Dissemination Area
MIZ: Metropolitan Influenced Zone
PCCF+: Postal Code Conversion File
2SFCA: Two-step floating catchment area
