# Supplementary material for: Accessibility of Ontario pharmacies offering COVID-19 vaccination by rurality, community material deprivation, and ethnic concentration: a repeated cross-sectional geospatial analysis
Source: BMC Public Health. 2025 Nov 12;25:3915. doi: 10.1186/s12889-025-24929-w (PMC12613703; doi:10.1186/s12889-025-24929-w)
Supplement: Supplementary file 1 — Supplementary Material 1. Appendix 1: Dates of analysis and their respective rationale. [file 12889_2025_24929_MOESM1_ESM.docx]

# Appendix 1: Dates of analysis and their respective rationale

| **Date** | **Rationale** |
| --- | --- |
| April 27, 2021 | All individuals aged 45 and over and who live in one of Ontario’s 114 hot spot communities were eligible to book a vaccine appointment through the provincial booking system |
| May 10, 2021 | 80 pharmacies locations offered Pfizer vaccine in Toronto and Peel and up to 60 pharmacies offered Moderna vaccine in Durham, Hamilton, Ottawa, Windsor-Essex, and York regions to individuals aged 18 and over. |
| May 12, 2021 | Select pharmacies in all 13 hot spot public health unit regions began administering Pfizer or Moderna vaccines to individuals 18 years of age and older in hot spot areas and to individuals aged 40 and over in non-hot spot areas. |
| May 23, 2021 | Youth aged 12 and over now eligible to book vaccine appointments |
| November 5, 2021 | Individuals aged 70 and older, health care workers and essential caregiver in congregate settings, individuals who received a complete series of a viral vector vaccine, and First Nations, Inuit and Metis adults and their non-Indigenous household members eligible to receive the 3^rd^ booster shot |
| November 22, 2021 | Quiet period (6,488 doses administered on this day vs. an average 23,989 doses in October) |
| December 13, 2021 | Individuals aged 50 years and over eligible to receive the 3^rd^ booster shot |
| December 20, 2021 | Individuals 18 years and older eligible to receive the 3^rd^ booster shot |
