# Supplementary material for: Accessibility of Ontario pharmacies offering COVID-19 vaccination by rurality, community material deprivation, and ethnic concentration: a repeated cross-sectional geospatial analysis
Source: BMC Public Health. 2025 Nov 12;25:3915. doi: 10.1186/s12889-025-24929-w (PMC12613703; doi:10.1186/s12889-025-24929-w)
Supplement: Supplementary file 2 — Supplementary Material 2. Appendix 2: Flowchart of pharmacy records into the study. [file 12889_2025_24929_MOESM2_ESM.docx]

# Appendix 2: Flowchart of pharmacy records into the study
